# Prevalence of depression during the SARS, MERS, and COVID-19 pandemics: A protocol for overview of systematic reviews

**DOI:** 10.1097/MD.0000000000022235

**Published:** 2020-09-18

**Authors:** Li Du, Ya-Min Chen, Ying Li, Wei Yuan, Jian-Shu Wang

**Affiliations:** a The Third People's Hospital of Lanzhou City; b Evidence-Based Nursing Center, School of Nursing, Lanzhou University; c Department of Obstetrics, the First Hospital of Lanzhou University; d Department of Cardiovascular Medicine, the First Hospital of Lanzhou University; e Department of bone and soft tissue oncology, Gansu Provincial Cancer Hospital, Lanzhou, China.

**Keywords:** COVID-19, depression, meta-analysis, systematic reviews

## Abstract

**Background:**

The outbreak of the novel coronavirus disease 2019 (COVID-19), caused by severe acute respiratory syndrome coronavirus 2 (SARS-CoV-2) infection, has emerged to be the biggest global health threat worldwide. COVID-19 marks the emergence of the third large-scale epidemic related to the coronavirus, after SARS-CoV in 2002 and Middle-East respiratory syndrome coronavirus (MERSCoV) in 2012. The pandemic has had a harmful effect on the public mental health, especially on depression. Increasing systematic reviews (SRs) of coronavirus were focusing on depression. However, the methodological quality of these SRs is unclear. Therefore, to evaluate and compare the normativity of report of SR, we conducted a comprehensive overview of depression during the SARS, MERS, and COVID-19 pandemics.

**Methods:**

Two independent reviewers will conduct comprehensively searches in PubMed, EMBASE.com, Web of Science, the Cochrane Library, Chinese biomedical literature database (CBM), Chinese National Knowledge Infrastructure (CNKI), Wan fang Database, Chongqing VIP (CQVIP). Reference lists of articles, gray literature, and conference proceedings will also be searched. We will extract the data and assess the methodological quality using the Assessment of Multiple Systematic Reviews-2 (AMSTAR-2) measurement tool and the Preferred Reporting Items for Systematic Reviews and Meta-Analyses (PRISMA) statement. General characteristics of the eligible SRs will be summarized and described. We will provide AMSTAR-2 and PRISMA assessments in tabular form for each review, the total percentage of each item will be calculated. Endnote X8 and EXCEL will be used.

**Results:**

Using the draft search strategy of databases, 8 SRs met the a priori criteria and were included. The overview of SRs will be published in a peer-reviewed journal.

**Conclusion:**

Our overview will be a comprehensive synthesis of the existing systemic review on depression with SARS, MERS, and COVID-19.

**Protocol Registration:**

INPLASY202080003

## 1 Introduction

Coronaviruses (CoVs) (order Nidovirales, family Coronaviridae, subfamily Coronavirinae) are enveloped viruses with a positive sense, single-stranded RNA genome.^[1]^ According to genetic and antigenic criteria, CoVs have been organized into 3 groups: α-CoVs, β-CoVs, and γ-CoVs. They can also infect humans and cause disease to varying degrees, from upper respiratory tract infections resembling the common cold, to lower respiratory tract infections such as bronchitis, pneumonia, and even severe acute respiratory syndrome (SARS).^[2–5]^ The outbreak of the novel coronavirus disease 2019 (COVID-19), caused by severe acute respiratory syndrome coronavirus 2 (SARS-CoV-2) infection, has emerged to be the biggest global health threat worldwide, which has now infected >15.2 million people and claimed >600,000 lives around the world. COVID-19 marks the emergence of the third large-scale epidemic related to the coronavirus, after SARS-CoV in 2002 and Middle-East respiratory syndrome coronavirus (MERSCoV) in 2012.^[6]^ World Health Organization (WHO) declared the epidemic as a high-risk Public Health Emergency of International Concern (PHEIC).^[7,8]^ Severe cases of COVID-19 can lead to heart, and respiratory failure, acute respiratory syndrome, or even death.^[9]^ In addition to the physical impacts, COVID-19 can have serious effects on people's mental health.^[10]^ A wide range of psychological outcomes has been detected during the virus outbreak, at individual, community, national, as well as international levels.^[11]^ A previous study demonstrated that early identification of individuals in the early stages of a psychological disorder makes the intervention strategies more effective.^[11]^ In the initial stage of the COVID-19 outbreak, studies from China showed that fear of the unknown and uncertainty can bring about the development of mental disorders such as stress, anxiety, depression, somatization, and adverse behaviors such as increased alcohol and tobacco consumption.^[12]^ The result of 1210 individuals in 194 cities of China showed that 16.5% of the participants were moderate-to-severe depressive symptom.^[12]^ At large, the pandemic has had a harmful effect on the public mental health, especially on depression, which can even lead to psychological crises.^[13]^

Systematic review is one of the most important evidence to guide clinical decision-making, which not only has important reference value for the formulation of clinical guidelines,^[14,15]^ but also can inform health care management and policy making levels by providing research-based responses to important questions about health systems.^[16]^ However, low-quality systematic reviews (SRs) can also mislead decision makers. Increasing SRs during the SARS, MERS, and COVID-19 pandemics were focusing on depression.^[11,17–24]^ However, the methodological quality of these SRs is unclear. Therefore, to evaluate and compare the normativity of report of SR, we conducted a comprehensive overview about prevalence of depression during the SARS, MERS, and COVID-19 pandemics.

## 2 Method

### 2.1 Design and registration

We will conduct an overview of SRs of depression during the SARS, MERS, and COVID-19 pandemics. Ethics approval is not required for overview of SRs. We will follow the Preferred Reporting Items for Systematic Reviews and Meta-Analyses (PRISMA) statement for reporting our overview.^[25]^ The study protocol has been registered with the International Platform of Registered Systematic Review and Meta-analysis Protocols (INPLASY) database (protocol number: INPLASY202080003, DOI: 10.37766/inplasy2020.8.0003).

### 2.2 Data sources and search strategy

Two independent reviewers will conduct comprehensively searches in PubMed, EMBASE.com, Web of Science, the Cochrane Library, Chinese biomedical literature database (CBM), Chinese National Knowledge Infrastructure (CNKI), Wan fang Database, Chongqing VIP (CQVIP). Reference lists of articles, gray literature, and conference proceedings will also be searched. Languages of the publications will be limited to Chinese and English. A draft search strategy using PubMed is presented in Table 1, whereas a draft search strategy using EMBASE.com is presented in Table 2.

**Table 1:**
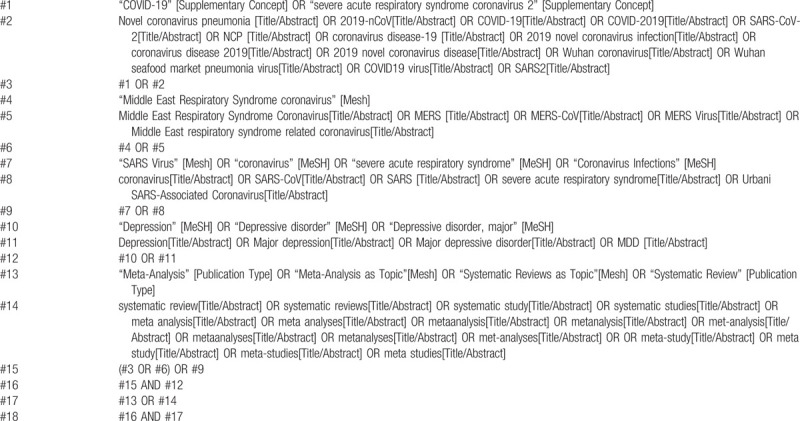
A draft search strategy using PubMed.

**Table 2:**
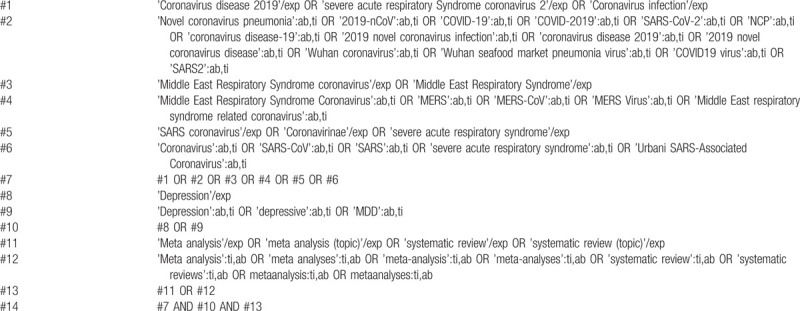
A draft search strategy using Embase.

### 2.3 Study selection

#### 2.3.1 Type of study

Systematic reviews and meta-analyses which take into account randomized controlled trials (RCTs), quasi-RCTs, as well as other studies (ie, cohort studies, case–control studies, cross-sectional studies), will be adopted.

#### 2.3.2 Inclusion criteria

1. Patients: Depression among the general population healthcare workers during the COVID-19 pandemic, and the patient has to be diagnosed with COVID-19. There were no restrictions on gender, age, or race.
2. Interventions: all interventions concerned.
3. Outcome: the prevalence of depression and sample size of depression. Depression will be defined by the score of relevant scales, such as Zung Self-Rating Depression Scale, Patient Health Questionnaire-9, Hamilton Depression Scale.
4. Published literature.
5. Studies whose full text was available.
6. Depression with organic diseases.

#### 2.3.3 Exclusion criteria

Exclusion criteria were literatures published repeatedly by the same author or with duplicate data; letter, scoping review, abstract; no peer-reviewed articles.

### 2.4 Data collection

#### 2.4.1 Literature screening

Records will be managed by EndNote X 8.0 (Thomson Reuters (Scientific) LLC Philadelphia, PA) software to exclude duplicates. At first, 2 reviewers independently (LD and YMC) will screen the titles and abstracts of the records to determine whether they meet the inclusion criteria. Then, the same 2 reviewers find the full text of all potentially eligible studies and assess the eligibility of each study according to the inclusion criteria. Disagreements are resolved by discussion or by a third reviewer (JSW), or the whole group members will join the discussion.

#### 2.4.2 Data extraction

Two main authors will independently collect data on study characteristics by using Microsoft Excel 2019 software to extract data from the included literature. The data extracted include: first author name, year of publication, country of first author, number of authors, journal name, funding, disease, outcomes (the score of relevant scales); types of included studies, number of included studies, samples, number and name of databases retrieved, supplemental literature search. Disagreements will be resolved by consensus or by discussion with a third reviewer (JSW).

#### 2.4.3 Quality assessment

Two reviewers (LD and YMC) will independently assess each included review by using the Assessment of Multiple Systematic Reviews-2 (AMSTAR-2) measurement tool and the (PRISMA) statement, for rigorous methodological quality and reporting quality.^[25,26]^ Arbitration by a third reviewer (JSW) was necessary for some fields. AMSTAR-2 is an update of AMSTAR, which can be used to appraise SRs of both randomized and nonrandomized controlled trials. The AMSTAR-2 tool consists of 16 items and has good face and content validity for measuring the methodological quality of SRs. Each item is described with “yes” (definitely done) and “no” (definitely not done), or “not applicable,” some items can be described as “part of yes.” Among them, entries 2, 4, 7, 9, 11, 13, and 15 are key items, and others are non-key items. The methodological quality is mainly according to the conformity of the key items, it is considered as 4 levels, namely “high," “medium,” “low,” “very low.” The PRISMA statement for reporting quality consists of a 4-phase flow diagram and a 27-item checklist, which includes items deemed essential for transparent reporting of systemic review. Each item of the PRISMA form was considered as “yes,” “incomplete,” or “no” and respectively scored as 1, 0.5, or 0 points for statistical analysis purposes. The total score of each questionnaire is divided by its maximum possible score to assess study quality. Study quality related to its PRISMA score as a percentage. Percentage was rated: very poor (<30%), poor (30%–50%), fair (50%–70%), good (70%–90%), and excellent (>90%).

### 2.5 Statistical analysis

General characteristics of the eligible SRs will be summarized and described, including the total sample size of a meta-analysis, interventions, and their effect size and related 95% confidence interval (CI). We will provide AMSTAR-2 and PRISMA assessments in tabular form for each review, the total percentage of each item will be calculated.

## 3 Result

### 3.1 Results of selected studies

**Figure 1:**
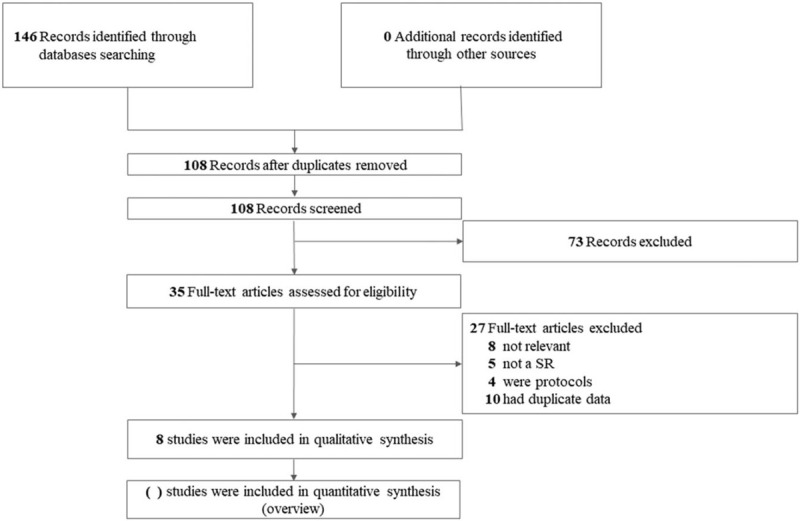
The flowchart of the screening process.

Using the draft search strategy of PubMed, EMBASE.com, Web of Science, the Cochrane Library, CBM, CNKI, CQVIP, 146 records were identified, of which 38 duplicates were removed and 108 records proceeded to title/abstract screening. The remaining 35 SRs were retrieved for full text for further eligibility, and 8 SRs^[11,18,21,23,20,24,27,28]^ met the a priori criteria and were included. The PRISMA flow chart of literature section is presented in Fig. 1.

### 3.2 General characteristics of included studies

We extracted the basic characteristics of some of the included studies. We included 8 SRs and SRs included cross-sectional study, interventional study, cohort study. Number of included studies was from 4 to 41 and included patients were from 1963 to 33,839. Population involved general public, health care workers, patients, isolation population. We also extracted the pooled prevalence (95%CI), assessment of methodological quality, whether meta-analysis conducted, whether subgroup analysis conducted, whether sensitivity analysis conducted. The details of characteristics of the included studies are summarized in Table 3.

**Table 3:**
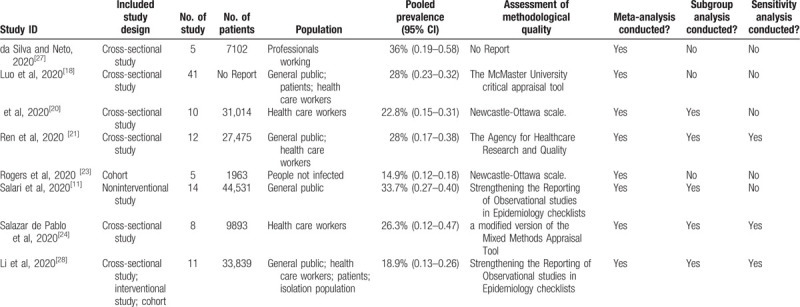
Basic characteristics of some of the included studies.

## 4 Discussion

Our overview will be a comprehensive synthesis of the existing SRs on depression with SARS, MERS, and COVID-19. To best of our knowledge, it will be the first overview in this filed.

In the discussion of our study, we plan to present the following sections: summary of main findings; comparison with other studies and opinions; implications for research and practice; interpretation of results; strengths and limitations; conflicts and funding; conclusion. We confirmed that the results of this overview will provide patients, physicians, and clinical researchers with information about the credibility of current evidence as well as future research direction.

## Acknowledgments

The authors thank the assistance of Dr. Jin-Hui Tian Dr., Ke-Lu Yang, and Dr. YA Gao to improve the methodology and quality of language in our manuscript.

## Author contributions

Li Du, Ya-Min Chen, Jian-Shu Wang conceived the study, developed the criteria, Li Du, Ya-Min Chen, Ying Li and Wei Yuan searched the literature, and analyzed the data. Li Du, Ya-Min Chen, Ying Li and Jian-Shu Wang wrote the protocol and revised the manuscript. All authors have read and approved the final manuscript.

**Conceptualization:** Li Du, Jian-Shu Wang.

**Data curation:** Li Du, Ya-Min Chen, Ying Li, Wei Yuan.

**Funding acquisition:** Li Du.

**Methodology:** Li Du, Ya-Min Chen.

**Software:** Li Du, Ya-Min Chen, Ying Li, Wei Yuan.

**Writing – original draft:** Li Du, Ya-Min Chen, Jian-Shu Wang.

**Writing – review & editing:** Li Du, Jian-Shu Wang.

## Abbreviations

Abbreviations: AMSTAR-2 = the Assessment of Multiple Systematic Reviews-2, CI = confidence interval, CoV = Coronavirus, COVID-19 = Corona Virus Disease 2019, HAMD = Hamilton Depression Scale (HAMD), LRTI = lower respiratory tract infection, MERS = Middle East respiratory syndrome), MERSCoV = Middle-East respiratory syndrome coronavirus, PHEIC = Public Health Emergency of International Concern, PHQ-9 = Patient Health Questionnaire-9, PRISMA = Preferred Reporting Items for Systematic Reviews and Meta-Analyses, RCT = randomized controlled trial, SARS = severe acute respiratory syndrome, SARS = severe acute respiratory syndrome, SARS-CoV-2 = severe acute respiratory syndrome coronavirus 2, SDS = Zung Self-Rating Depression Scale, SR = systemic review, URTI = upper respiratory tract infection, WHO = World Health Organization.
